# Supplementary material for: A surface morphology-based inference method for the cell wall elasticity profile in tip-growing cells
Source: PLoS Comput Biol. 2026 Apr 2;22(4):e1013532. doi: 10.1371/journal.pcbi.1013532 (PMC13082730; doi:10.1371/journal.pcbi.1013532)
Supplement: Attachment — Submitted filename: PCOMBIOL-D-25-01862_review.pdf [file pcbi.1013532.s021.pdf]

Summary

The manuscript *PCOMPBIOL-D-25-01862* entitled "A surface morphology-based inference method for the cell wall elasticity profile in tip-growing cells", by Rholee Xu, Luis Vidali, and Min Wu addresses the topic of tension and effective bulk elastic modulus inference for tip-growing walled cells. The authors present a novel computational framework, relying on surface triangulation, to estimate the spatial distribution of the effective bulk elastic modulus in the cell wall of tip-growing cells. This approach integrates the acquisition of marker points—fluorescent beads or quantum dots—and their 2D Delaunay triangulation following stereographic projection. Data are acquired before and after osmotic treatment, enabling the construction of a discrete deformation mapping (and its gradient) between both configurations. A method for estimating the extrinsic curvature of the deformed configuration is also introduced. By applying a generalized Young-Laplace law, the authors express the stress values in meridional and circumferential directions as functions of previously computed geometric quantities. The mechanical model assumes axisymmetry of the cell wall surface.

This work focuses exclusively on simulated data, generated numerically, to mimic the shape of *Physcomitrium patens* moss cells. The objective is to benchmark the method and evaluate its sensitivity with respect to two main parameters: noise in the marker positions and the resolution of the generated triangulated mesh.

Ultimately, the authors propose confidence maps (Fig. 8) for the computed "endpoint bulk modulus ratio" as a function of marker position noise level and triangulated mesh resolution.

Main reported results

The workflow enables the generation of triangular surface meshes from intensity 3D images of cell walls labeled with point-like fluorescent markers (Fig. 1). From these meshes, geometrical quantifiers of surface shape, e.g., extrinsic curvature (Fig. 2) and deformation between two configurations of the same mesh, are computed. A method for generating axisymmetric artificial surfaces to test the procedure is also described (Fig. 3). Tests are performed under mechanical equilibrium, assuming various spatial distributions of the effective bulk elastic modulus. The authors then apply their inference method to these test cases and retrieve the spatial distributions of various mechanical quantities—elastic stretch ratio, curvature, tension, and effective bulk modulus—with good precision (<4% relative error, Fig. 4). Realistic measurement noise is incorporated into the artificial surfaces, showing the sensitivity of the inference process to both noise and mesh resolution (Fig. 5). Averaging across multiple cells (n=10) improves inference precision (Fig. 6, Figs. S8–S12). A systematic exploration of the parameter space for constant and varying rigidity is presented. The authors monitor the inferred endpoint measure (ratio $K_{\mathrm{side}}/K_{\mathrm{tip}}$) for various noise and resolution levels, interpolating results across the relevant domain. This highlights potential overestimation of endpoint measures, especially for high values of the side bulk modulus (Fig. 7, Fig. S13). Absolute and relative error on the endpoint measure is estimated for two cell profiles inspired by real cells (*P. patens caulonema & chloronema*), with reported confidence intervals for the endpoint inference (*e.g.,* high noise Fig. 8, medium Fig. S15, and low Fig. S14).

Major Comments

1. The manuscript would benefit from further attention to writing and structure. Some sections might be better suited for supplementary material, such as the derivation of the extrinsic curvature estimation (lines 117–142), results from the two methods to quantify surface area changes (line 371), and the analysis of the sphere-based inference method (lines 346–364). Though these details are relevant for the scope of the authors' work, they obscure the main message and may be more appropriately presented as supplementary information.

2. Several sentences and expressions require clarification for non-native English-speaking readers. For example:

- Line 252: The title of this paragraph is unclear; what is the function f?
- Line 262: “The last conversion to the outline model” is ambiguous.
- Line 426: The meaning of “knee value” requires definition.

3. Some mathematical descriptions could be clarified further:
   - Line 141: In which configuration is curvature computed? The deformed configuration is presumably intended, but this should be specified.
   - Line 173, equations 7 and 8: Shouldn't there be a $1/(\lambda_s \lambda_\theta)$ factor for the first term on the right-hand side?
4. It would be advisable to consider making source code openly available in accordance with FAIR principles. While proprietary software is used (this is not ideal, but I could live with that), interested readers and potential users should at least have access to scripts and files needed to reproduce the presented results from an online repository.
5. The stereographic projection-based triangulation method is interesting but departs from standard algorithms such as 3D Delaunay triangulation. The rationale for preferring this approach should be discussed. Are such algorithms (3D Delaunay) not available in Matlab? Is there a risk of amplifying point cloud noise through stereographic projection?
6. It is also not clear to me how vertex pairing between configurations is ensured. Could you elaborate on this?
7. Currently, the method is tested only on synthetic data. A case study on real datasets would substantially strengthen the manuscript.

Minor Comments

- Line 170: It would be instructive to evaluate the influence of different elastic potentials (besides Neo-Hookean) on bulk modulus estimation. Indeed, the Neo-Hookean model might not be the best-suited one to describe plant cell walls. Even if the ones considered here can be assumed isotropic, I wonder about their compressibility.
- Line 182: Equations 9 and 10 may be replaced with those for bulk and shear moduli expressed as function of the geometrical descriptors and obtained by combining equations (Eqs. 5–8).
- Line 230: How is the tip position of the cell determined?
- Fig. S4C: Indicate which curves correspond to circumferential and meridional stretches.
- Line 297: Null Poisson's ratio hypothesis may be bold, given the material properties at stake.
- Lines 273–291, Fig. 3: It would be valuable to test artificial surfaces lacking axial symmetry (e.g., by deforming the point cloud orthogonally to the main axis).
- Fig. 3D: The normalized displacement field noise appears high ($\sim 10\%$). It would be informative to report noise level on the deformation gradient ($\boldsymbol{F}$), in order to estimate the signal/noise ratio on the stretch estimation, central to the inference pipeline.
- Lines 300–302: Origin of expressions for Y_{side} and Y_{tip} should be documented.
- Line 477: Maybe I missed the information, but how many samples did you take on the grid you mention on the $\{K_{side}, K_{tip}\}$ space?
- Line 487: I find the term “false-positivity” hill-suited to the context, for there is no binary (true/false) results here. Wouldn't the term “over-estimation” be more relevant?

- Fig. 7: Unusual color map orientation (lowest values at the top), this is a bit surprising.
- Supp. Mat S1: In Eq. 3, should the denominator be F^0_{2D}?
- Section 1.4 refers to Fig. S2C but only two subpanels are in Fig. S2.
- Supp. Fig S7: Caption switches between tension and elastic stretch for panels B and C.
- Sensitivity analysis is a notable strength; authors may elaborate further, *e.g.,* how triangle shape and size affect sensitivity. Key figures could be moved to the main text.

Personal option

The manuscript *PCOMPBIOL-D-25-01862* proposed by Rholee Xu and coworkers presents a substantial work and I particularly enjoyed reading the developments of the sensitivity analysis proposed in Supp. Mat. S1, as well as the noise *versus* resolution analysis.
Nevertheless, in its present form, the manuscript does not — to my opinion — meet the publication standards of PLOS Computational Biology, primarily owing to three concerns: *(i)* The reliance on proprietary software and lack of open-source code. *(ii)* The absence of demonstration on experimental datasets. *(iii)* The reliability of the stereographic projection-based triangulation, which should be if not demonstrated at least discussed properly.
Finally, a substancial re-writing effort should be consider to make the text more accessible to a wider audience.
Addressing these points will greatly enhance the manuscript's scientific value.
