# Supplementary material for: A surface morphology-based inference method for the cell wall elasticity profile in tip-growing cells
Source: PLoS Comput Biol. 2026 Apr 2;22(4):e1013532. doi: 10.1371/journal.pcbi.1013532 (PMC13082730; doi:10.1371/journal.pcbi.1013532)
Supplement: Attachment — Submitted filename: Rebuttal Letter Final.docx [file pcbi.1013532.s022.docx]

We would like to thank the reviewers for their thoughtful and comprehensive feedback on our manuscript. Their comments and suggestions have helped us greatly in improving the manuscript. We have compiled itemized responses to their comments below. All changes in the main text and supplemental text have been marked in red.

***Reviewer 1 – Major Comments***

*1. I don't understand the overall logic of the protocol. Right from the start (i.e. in the abstract), the authors state that "Previous work is based on the wall meridional outline on the assumption the cell is axisymmetric, an idea that does not align well with the reality of the cells.". The observed deviation from perfect axial symmetry is the motivation for the new experimental protocol based on image stacks and the positioning of cell surface markers in 3D without assuming axial symmetry. The puzzling part, for me, is that once the curvature and strains are computed on a 3D triangulated surface, these variables are then projected onto an axisymmetric shape (Fig. 4) and they are ultimately averaged between several cells to reduce noise (Fig. 6). The proposed method is thus technically challenging, because it starts with the localization of markers in 3D space; and yet it concludes with a "projection" of the variables on an axisymmetric surface. In my opinion, the authors take a more challenging path to arrive at the same endpoint as all prior protocols. Moreover, inspection of the 3D reconstructions of an actual cell in Fig. 2F and G would suggest that these reconstructions are too noisy to be useful on their own. The projection onto an axisymmetric surface seems almost necessary for a quantitative interpretation of the cell surface curvature and strain. So, why not start with the axisymmetric geometry?*

*From a biophysical standpoint, a 3D reconstruction of the cell surface is most useful if the cells under study deviate SYSTEMATICALLY from axial symmetry. That would be the case for cells that are growing along a well-defined 2D curve or some 3D helical path. While some tip-growing cells do show these behaviors, most tip-growing cells are surprisingly good at maintaining their growth direction and shape. As a result, the small deviations from axial symmetry tend to cancel out if a cell is tracked for some time or if many cells are compared among themselves. Without clear, repeatable, deviations from axial symmetry, it is not obvious what is the benefit of a 3D reconstruction.*

*Given the observations stated above, I'm confused as to why the protocol presented is superior to measuring the cell curvature and strain directly on a median section of the cell. Experimentally and computationally, the standard protocol is easier to implement and potentially more robust in its estimate of the curvature and strain because it relies on the x,y-position of the cell contour and surface markers, and leaves out the z position whose spatial resolution is far inferior to the x,y-position in most microscope setups. I'm thinking especially of the pole of the cell where a robust 3D reconstruction is particularly challenging when the cell is observed on its side. I would therefore ask the following question to the authors:*

*What are the concrete ways in which their protocol is superior to earlier protocols where axial symmetry is assumed from the start?*

*Finally, I should note that the lack of a perfect axial symmetry has been taken into account in some prior work. This was done by computing the curvature and other variables independently on the "left side" and "right side" of the cell. In all the cases that I can readily remember, the slight asymmetries in cell shape have little impact of the value of the variables that are computed.*

**Response:** Thank you for the detailed comments of your concerns with the 3D reconstruction. The main difficulty we faced, and the reason behind the 3D reconstruction, was the calculation of the elastic stretch ratios. With the measurement of the elastic deformation, our experiments did not involve tracking the active growth of the tip-growing cell. Previous works with an axisymmetric assumption have only measured the wall strain rates by tracking the bead movement while the cell is growing. We encountered experimental limitations with the fluorescent bead coverage as well as observations of bead rotational movement after plasmolysis (see Figure 1 below). Due to the unpredictable rotational movement after plasmolysis, we found there was no systematic way to track the changes along the meridian, unless we did have perfect axial symmetry. This is where we may have misled what the motivation of our method is, by stating that it was the observed deviation from axial symmetry. The cell surface does need to be close to axisymmetric in order to calculate the optimized meridional and circumferential directions to then use the axisymmetric model. These points have been added and re-written in the main text, specifically in the Abstract, Line 54 and Line 194.

While we have seen two previous protocols also measuring changes after cell plasmolysis, both protocols did not measure the explicit change of arclength along the tip between the two configurations [1, 2]. As for the curvature and tension method, we don’t claim that our protocol using the 3D reconstruction is better than previous protocols. However, for the goal of the elastic moduli gradient along the tip of the cell, we need the tension information to be compatible with the elastic stretch ratios. Therefore, we also used the wall surface to calculate those parameters. In this sense, our protocol is a more systematic way of getting to the spatial elastic moduli without assuming perfect axial symmetry.

As for the z-position noise, we believe the software RS-FISH is quite robust in locating the center of the bead using their radial symmetry technique which outputs a sub-pixel z-position. In addition, we also addressed the noise in the z-position from estimations in experiments (Figure 3D main text and Line 294). This noise was added to test our method on the simulated cells. We do not have sub-pixel z-position resolution of the wall outline, which may contribute to some of the noisiness seen in the reconstructions in Fig 2F. But by using the entire available outline, we can recover the trend of curvature at the resolution of the triangulation. We will show in the point below the comparison of curvature calculation using the 3D reconstruction, and curvature calculation using the median outline.

*2. On a related note, I would urge the authors to perform a direct quantitative comparison between their protocol and one based on the median section of the cell. It seems the authors already have the data to do a comparative study for the meridional curvature. As far as I can tell, Fig. S3 was prepared by extracting the contour from median sections of several cells. These data could be compared to the curvature that it arrived at by projection of 3D reconstructions. I would suggest that the authors do the same for the elastic strains. These comparisons would go a long way in justifying the use of the new protocol.*

**Figure 1: Demonstration of fluorescent bead rotation after plasmolysis**: Three examples of bead movement after plasmolysis. For all images, the view shows the x-y plane of the cell and is stopped where the software has highlighted the center of the bead (in green). The red box in the corner shows the z-position of the current view.

**Response:** Thank you for this suggestion. First, we want to point out that our inference of parameters in the simulated cells shown against the ground truth is a way of comparing our protocol to a “perfect” measurement using the median section. For the results with no noise, see the old Fig 4 (new Fig S9), and old Fig S7 (new Fig S10). In Deng et al., where the marker point inference scheme was first introduced, measurements made from the wall outline are also compared against the ground truth [3]. Like in other outline protocols the meridional curvature was calculated using $\kappa_{s}\left( s \right)={d\alpha}/{ds}$. The meridional elastic stretch ratio is $ds/ds_{0}$, where $s_{0}$ represents the arclength in the plasmolyzed configuration. The results with and without noise emphasize the impact of the choice of $ds$ in outline methods, analogous to the choice of triangulation size in our protocol. We have seen how there can be an over-estimation of tensions at the tip due to noise, but also $ds$ (see Figure 2A below). Similarly in our protocol, with a large triangulation, the meridional curvature can sometimes be underestimated (see Figure 2B below). However, at the scale of the tension and eventual bulk modulus, this error is not significant. The results of this using the same triangulation is in the old Fig S7 (new Fig S10). In general, it was reassuring to see that we reached the same conclusion as our outline method inference. When $ds$ or triangle size is small, it is more accurate but more susceptible to noise than a larger $ds$/triangle size.

Originally our plan was to replicate the marker point inference on the outline as done in Deng et al. But, as stated previously, with our experimental capabilities, we are not able to measure the elastic strain using just the median outline of the cell. In order to make the curvature/tension calculation compatible, we used the wall surface. Since the concern is the comparison using experimental data, we attempted to measure the meridional curvature using a median outline of the cell. Due to time, we did not fully replicate methods used in previous papers. Instead, we extracted a median outline through the center of the cell and fit a hyphoid curve on both sides separately to obtain a smooth result (see Figure 2C below). We calculated the meridional curvature of the two sides separately using the equation $\kappa_{s}\left( s \right)={d\alpha}/{ds}$ where $ds$ was arbitrarily decided based on the curve discretization. We first observed that our results along the arclength of the cell were comparable with other published results [4] (see Figure 2D below). When we converted the results back to the local angle, we compared that with our results using the surface method (see Figure 2E below). As we can see, the results are within the right range but have differences due to the method of curve fitting for the outline, and triangulation size for the surface method. As stated above, since we did use a larger triangulation in our experiments (see new Fig 6A), this may explain some of the lower inferences of $\kappa_{s}$ at the tip using our method. If the goal was just the curvature, we would use a medium or small triangulation, which is accurate to the ground truth, justifying its comparison to outline based methods. For experimental measurements of the elastic moduli and more uncertain triangulation sizes, our manuscript justifies the use of our protocol.

**Figure 2: Meridional Curvature Comparison**. **A.** Figure 7 from Deng et al. 2022. Tension results are calculated from the curvature. The separation at the tip is a direct result from the separation at the tip of the curvatures. **B.** Curvature results on a simulated cell with different triangulation sizes and no noise. **C.** Example experimental median outline used and corresponding fitted hyphoid curve. **D.** The result of meridional curvature along the arclength of the cell. **E.** For a selection of experimental cells, the meridional curvature calculation using a single median outline of the cell is plotted in black along the local angle. The two black lines represent results from each side of the cell. The red error bar line represents the meridional curvature calculated through the surface morphology method.

*Also, the addition of more biological data would increase the appeal of the paper. As it stands, the focus of the manuscript is on simulated data instead of data from real cells.*

**Response:** We have now added experimental data from our *P. patens* cells. This has been incorporated into the Results section of the main text (Line 440) and summarized in the new Figure 6. Additional clarification on the application of the method on the experimental data can be found in the Methods on Line 66 and Section 1.2 in S1 Text.

*3. I also several questions regarding the data acquisition protocol described at the beginning of the Methods section.*

*3.1. I believe the cell wall is fluorescently labeled with calcofluor and this information was used to make the reconstruction of Fig. 2F. It is not clear, however, whether the beads that are used to track the elastic strains are positioned in 3D such that they sit on the surface defined by the cell wall. Stated differently, a 3D reconstruction can be achieved by connecting the fluorescent beads into a triangulated surface (as seen in Fig. 2A) or by stacking the fluorescent wall outlines as in Fig. 2F. My question is whether these two sources of 3D information are forced to be compatible with each other. The "wall contour" approach is obviously the richest in terms of positional information. It would make sense to confined the beads to an interpolated surface defined by the wall outlines.*

**Response:** Thank you for addressing this point, which we did not make clear in the original manuscript. With the added addition of our experimental data, we have added more information to this aspect of the procedure. Like your point states, we originally noticed this issue and addressed it by projecting the marker point locations to the wall contour (see new Fig S1E, and also photo on the right). On average these projections were within 2 µm. Any projections that extended more than 5 pixels in the Z-direction were removed, and visual inspection was done on the projections to ensure that none looked inaccurate. This was done for both the turgid and unturgid marker points and wall outline. Descriptions of this procedure have now been added to Section 1.2 in S1 Text.

*3.2. I'm confused with one aspect of the stereographic projection. I understand the principle of the Delaunay triangulation and the need for a well-defined 2D surface for the triangulation to be computed. If the interpolated surface mentioned above was computed, then a Delaunay triangulation could be computed on it. Instead, the authors chose a mixed stereographic projection of the cell surface markers onto a plane to implement the triangulation. The topology obtained on the plane is then used to triangulate the cell surface. I would like to know if the nice geometrical properties of the Delaunay triangulation are preserved when the triangles are mapped back onto the cell surface. I don't remember clearly all the properties of a proper Delaunay triangulation but it seems that one of them is that the triangles are made as "equilateral" as possible given the set of points. My concern is that the large geometrical deformation imposed by the stereographic projection could lead to a triangulation that is not optimal once it is applied to the curved cell surface. Did the authors investigate that? Would it not be possible to perform the Delaunay triangulation directly on the interpolated cell surface obtained from the calcofluor staining?*

**Response:** Thank you addressing this point, we have aimed to further show the advantages of the stereographic-projection method. There is a way to implement the “delaunay” function in MATLAB on a 3D point cloud, however it outputs a tetrahedron mesh. Its implementation and refinement are not as elegant as the 2D Delaunay, and we found that it does not create more compact triangles. To compare, we recovered the surface triangulation of the 3D Delaunay method using a convex hull function (see Figure 3A below). Compared to our original triangulation method for all three triangulation sizes, we saw that our method created more compact triangles (see Figure 3B below). We quantified this using a compactness score: $4\pi A/P^{2}$, where A is the triangle area and P is the triangle perimeter. As we can see, the 3D method has more slender triangles in yellow with scores closer to 0. Given that a triangle will have a maximum score of approximately 0.6, our projection method led to more compact triangles with scores closer to 0.6 (see Figure 3C below).

But it is true to your point, that the original angle properties of the flat 2D triangulation will be different once it is projected onto the 3D surface. We have addressed this in two ways. First, we have already implemented an analysis described in the “Triangle Error Analysis” section of S1 Text, which we found to remove slender and small triangles sensitive to perturbations. We displayed the triangles that would be removed by this algorithm highlighted in red, which demonstrates that our method helps preserve more triangles to work with. After filtering, we also showed the resulting histogram of triangle compactness in red, confirming that the filtering generally shifts the distribution to the right (see Figure 3C below). Additionally, for all our experimental triangulations, we manually check the output of the automation and can remove triangles that do not match the cell surface (This part of the process is explained in Section 1.2 of S1 Text). Based on our observations of multiple triangulation outputs on simulated cell surfaces, we believe the stereographic projection method is optimal specifically for surfaces shaped like tip-growing cells.

**Figure 3: Comparison of triangulation techniques: A.** Surface triangulation of 3D Delaunay algorithm. **B.** Stereographic-projection method presented in our main text, on the same set of marker points. For both **A.** and **B.**, the triangle color indicates the value of its compactness score. The triangles outlined in red are triangles that would be filtered out using our sensitivity analysis algorithm. **C.** Histogram of compactness score for the two triangulations. The histogram outline in red is the result after filtering out the triangles shown in red in **A.** and **B.** respectively.

*3.3. A final point, it is not clear how the equivalent points on the turgid and plasmolyzed cell are matched with each other. It can be a very tedious task, especially if the number of markers is large and the change in cell geometry is substantial. Was the matching done manually?*

**Response:** Thank you for addressing this confusion. We have now added a better demonstration of how this is done in the new Fig S1C (see photo to the right also). The matching is done manually by plotting the two marker point sets together in Matlab. Sometimes, when the pairing is unclear, we add a global displacement of one set of marker points to aid the visual matching. For marker points that may have multiple potential matches, we further confirm the correct pairing by checking the raw data of the marker points. In ImageJ, we can visually confirm the location of the fluorescent bead before and after the deformation based on how the bead looks and its surrounding beads. If there is any uncertainty after visual confirmation in the raw data, we will not use that marker point position. This explanation has also been added to the Methods in Line 74, and also in more detail in Section 1.2 of S1 Text.

*4. Finally, the text should be checked thoroughly. There are many sentences that are poorly phrased, obscure, or plainly wrong. I'm listing some issues below but I must confess that I gave up after a while. Try to eliminate or shorten the "noun chains" that appear in the manuscript. For example, the expressions "Marker point triangulation scheme", "fluorescent bead covered surface", "cell wall surface marker points" are hard to read.*

**Response:** Thank you for the suggestions, we have rephrased or addressed the bulleted points below. We also aimed to rewrite additional parts of the text which may have been poorly phrased.

*Abstract*

- *"Plant cell morphology and growth are essential for plant development and adaptation, in particular the cell wall material deposition and rearrangement. " This sentence is awkward. I would rephrase it.*
- *"As the wall is extended due to turgor pressure, the wall mechanical response, specifically the wall’s elastic properties, are not well understood. " This sentence is nonsensical.*
- *"Previous work is based on the wall meridional outline on the assumption the cell is axisymmetric, an idea that does not align well with the reality of the cells. " Please clarify what you mean.*
- *"Instead, we developed a way to triangulate the surface of the cell using marker point locations that could be achieved experimentally with fluorescent labeling. " Rephrase.*
- *"a mechanical model is used to measure the tensions and surface bulk modulus distribution " Models are not used to "measure".*
- *"We simulated moss tip cells from experimental Physcomitrium patens plants to challenge the method with noise estimated from experiments. " This sentence is nonsensical.*
- *"causing false-positive gradient results " Please clarify what you mean.*
- *"understanding tip cell growth " The expression "tip cell" appears in many places. It is not clear if you are talking about the "cell's tip" or a "tip-growing cell".*

*Author summary*

- *"Their growth and morphogenesis are tightly regulated processes involving cell wall addition and rearrangement, which aim to reduce the wall stress originating from the cell’s internal turgor pressure. " wall addition and rearrangement is not "aimed" at reducing the wall stresses.*
- *"We simplify this cell growth system by first studying the cell wall’s elastic properties that are present without active growth. " This sentence is awkward. Moreover, the elasticity of the cell wall is present whether or not growth is occurring. Your own "Dual-Configuration Model" says so.*
- *"We use a surface triangulation method derived from marker point tracking to measure the spatial elasticity along the tip cell " Rephrase.*
- *"Our results suggest that this inference method can reliably measure a cell wall elasticity gradient under combined geometric and mechanical conditions that create large enough elastic strain at the tip. " Clarify "large enough". 5%? 10%? 50%?*

*Introduction*

- *line 7 "those mechanics differ in particular areas along the cell surface. " "those mechanics"? Do you mean "mechanical properties"?*
- *line 19 "The scale of single tip-growing cells make it difficult to measure wall mechanics, especially at discrete spatial locations. " SMALL scale (give the size) makeS. What do you mean with "measure wall mechanics". Do you mean to "measure the wall's mechanical properties"*
- *line 22 "In tip-growing cells, the use of micro-indentation has been a common tool used to measure local mechanical differences in pollen tubes [13–15]. Due to the geometrical orientation of the probing, the measurement may not provide a direct measurement of the mechanics relevant to wall expansion " The first sentence should be rephrase. Also both sentence have awkward repetitions of words: use(d) and measurement.*
- *line30 "The models note that a steep gradient of mechanical properties ..." "The models note ..."? I would say "Results from the models indicate ..."*
- *line 32 "An alternative to Lockhart is the idea that the wall growth comes from the combination of two actions, the first ... reversible elastic deformation ... the second action involved is the irreversible wall expansion" The view proposed is NOT an alternative to Lockhart because the very first equation in Lockhart's paper (Eq. 1) is a statement that the observed change in cell length is the sum of reversible and irreversible deformation of the wall. The reason why wall elasticity does not appear in the Lockhart Equation is because it is the relation for steady-state growth, i.e. when pressure is not changing in time.*
- *line 43 "Importantly, the results showed a gradient of properties within one order of magnitude. " What properties? What gradient?*

**Response:** Thank you, these points in the Abstract, Author Summary, and Introduction have all been rephrased or rewritten.

*Methods and Results*

- *line 63 "cell wall surface marker points " how about "cell surface markers"*
- *line 64, the six lines starting at lines 64 include details that are specific to the methods of this paper, which is the purpose of the methods section, and methodological details that are not used in this paper. "In experiments, the marker points can come from the adhesion of fluorescent beads or 65 quantum dots, for instance [9,20,23] ". "The unturgid state can be obtained by removing turgor pressure 68 with an osmoticum change [23,29] (Fig 1A bottom) or laser ablation [27,30]." I would eliminate from the methods section the mentions of alternative protocols and focused instead on explaining clearly what was done.*
- *line 117 "The standard calculation of principal curvatures in the outline model and previous works is denoted by: κ ̄ s = dα/ds and κ ̄θ = sin α/r ". I don't understand*
- *line 119 "On the wall surface, the s and r parameters are hard to define even in the optimal sense. " Clarify.*
- *line 157 "In the axisymmetric system, the wall properties can be defined on two orthogonal directions along the circumferential (θ) and meridional (s) coordinates. " On an axisymmetric geometry, all variables can be expressed in terms of arc length position (s) because there is no variation in the circumferential direction.*
- *line 170 "For simplicity, we assume the 2D nonlinear Hookean constitutive law relates the tensions with the elastic stretch ratios through two material properties, the surface bulk and shear modulus, K ̄ h and μ ̄ h, respectively" Rephrase*
- *line 181 "Subsequently, K ̄ h and μ ̄ h can be measured by rearranging Eqs 7 and 8: " Parameters are not "measured" by rearranging equations.*
- *line 193 "Due to the isotropic nature of the tip cell, this will always occur at the tip." Here, I assume "tip cell" means "pole"*
- *Figure 2 and Figure 5, the colormaps are almost opposite. In Fig. 2, red is low and blue is high. In Fig. 5, blue is low and yellow is high.*

**Response:** Thank you for these corrections. Besides the last point, the other points have been changed or clarified. For the last point, since Figure 2 and Figure 5’s colormaps are different (“turbo” versus “parula”), and they are plotting different values we did not notice this. Visually, we think Figure 2 looks better where most of the cell side is red instead of blue, so we have not changed the colormaps.

*Discussion*

- *line 522 "The spatial cell wall elasticity in tip-growing cells is one determining factor of where plant cell walls grow, making them an important measurement for tip growth models. " What is "them" referring to?*
- *line 525 "By using a surface triangulation method to connect marker points across the cell surface, we were able to adopt the axisymmetric mechanical model to measure the wall elastic moduli (Fig 1, 2). " Do you mean "adapt"?*
- *line 570 "that a courser spacing of marker points " Do you mean "coarser"?*
- *Finally, the movies in supplementary material do not seem very useful. Static plots would be more easily compared.*

**Response:** Thank you, we have corrected these points.

***Reviewer 2 - Major Comments***

*1 . The manuscript would benefit from further attention to writing and structure. Some sections might be better suited for supplementary material, such as the derivation of the extrinsic curvature estimation (lines 117-142) , results from the two methods to quantify surface area changes (line 371) , and the analysis of the sphere-based inference method (lines 346–364) . Though these details are relevant for the scope of the authors’ work, they obscure the main message and may be more appropriately presented as supplementary information.*

**Response:** Thank you for these comments. We have moved most of the details of the curvature estimation to the new Section 1.3 in S1 Text. The section in the main text is still there so that we can reference Figure 2C-F, but it is considerably shortened. In the Results section, we kept the inference with no noise using the sphere-fitting and marker point method because we believe it helps transition to the parameter sensitivity study with noise. Particularly, the transition using Figure 5A to the rest of the Figure 5B-D. However, we did shorten them to get to the main message faster and moved the old Figure 4 to the new Fig S9.

*2. Several sentences and expressions require clarification for non-native English-speaking readers. For example:*

- *Line 252: The title of this paragraph is unclear; what is the function f?*
- *Line 262: “The last conversion to the outline model” is ambiguous.*
- *Line 426: The meaning of “knee value” requires definition.*

**Response:** Thank you, these parts have been rewritten for more clarity. The title of the paragraph has been changed to “Computation of mechanical properties on the wall surface” on the Line 221. We have also corrected the phrase “The last conversion…” on the Line 231. Lastly, we added the definition of “knee value” in Line 404.

*3. Some mathematical descriptions could be clarified further:*

- *Line 141: In which configuration is curvature computed? The deformed configuration is presumably intended, but this should be specified.*
- *Line 173, equations 7 and 8: Shouldn’t there be a 1/(λ_s_λ_θ_) factor for the first term on the right-hand side?*

**Response:** We compute the curvature on the unturgid configuration. This has now been specified in Line 130 of the main text. As for the second point, if we are understanding your question correctly, the $\frac{1}{\lambda_{s}\lambda_{\theta}}$ factor appears when $\bar{K}$, the 3D elastic modulus, is considered. We can observe that this factor gets absorbed into the surface elastic moduli $\bar{K}_{h}$ in the relation: $\bar{K}_{h}=h_{0}\frac{1}{\lambda_{s}\lambda_{\theta}}\bar{K}$, where $h=\frac{h_{0}}{\lambda_{s}\lambda_{\theta}}$ due to 3D incompressibility where the thickness stretch ratio reciprocates the area stretch. Since $\bar{K}_{h}$ is considering the distributions of the wall thickness in the two configurations, we assume that the surface elastic modulus is rescaled by $h$: $\bar{K}_{h}=h\bar{K}$ (see Line 163 in the revised main text). When there is enough information on the distributions of $h$ and $h_{0}$, this part of the equation can be added. To explain this in the main text as well, we have added the last line of this explanation to Line 163.

*4. It would be advisable to consider making source code openly available in accordance with FAIR principles. While proprietary software is used (this is not ideal, but I could live with that), interested readers and potential users should at least have access to scripts and files needed to reproduce the presented results from an online repository.*

**Response:** We had previously included a GitHub link to the code but did not reference it in the main text. To further improve the accessibility of the code now that we have added more experimental descriptions, we have added an in-depth demonstration of how to apply the code to experimental data. Our hope is that people use our code to make these measurements themselves, so we have not tried to limit any of the code/software used. The updated repository and demo can be found here: <https://github.com/rholee-xu/surface-model/tree/main>

*5. The stereographic projection-based triangulation method is interesting but departs from standard algorithms such as 3D Delaunay triangulation. The rationale for preferring this approach should be discussed. Are such algorithms (3D Delaunay) not available in Matlab? Is there a risk of amplifying point cloud noise through stereographic projection?*

**Response:** Thank you for addressing this deviation from the standard algorithm. We were aware that the same software in Matlab that we used (the “delaunay” function), could also be used to perform a 3D Delaunay triangulation. However, given a point cloud, the initial output given is a tetrahedron mesh. The tip surface can then be recovered using the convex hull function on the tetrahedron (see Figure 1A, below). However, further reading on the 3D algorithm showed that, mathematically, the 3D delaunay doesn’t guarantee desirable compactness of the triangles. Given that our method is made specifically for shapes with the geometry of a tip-growing cell, we believe our 2D triangulation method on the projected surface is optimal.

We confirmed this using our synthetic cell set-up with the three triangulation sizes. We applied the Polsby–Popper score, which is a measure of shape compactness. The formula for this is: $4\pi A/P^{2}$, where A is the triangle area and P is the triangle perimeter. For each triangle, the color scheme denotes this value. The maximum value a triangle can have is approximately 0.6, and less compact triangles in yellow have scores closer to 0. For all three triangulation sizes, the 3D Delaunay method has fewer compact triangles (see Figure 1C, below).

Our method will still create slender triangles, but we have used the algorithm explained in the “Triangle Error Analysis” section in S1 Text to remove triangles sensitive to perturbations. Our filtering algorithm removes triangles one standard deviation above the mean of $\log\Gamma$. We highlighted which triangles would be removed in red for both cases (Figure 1A,B below, red triangles). After filtering, we also re-summarized the histogram of compactness score (Figure 1C below, red outline). In general, we see that the filtering shifts the distribution to the right. Overall, the stereographic projection leads to less noise because there will be less triangles removed through filtering, and therefore more data available. The projection does not change the position of the final marker points, so it shouldn’t amplify point cloud noise in that sense.

**Figure 1: Comparison of triangulation techniques: A.** Surface triangulation of 3D Delaunay algorithm. **B.** Stereographic-projection method presented in our main text, on the same set of marker points. For both **A.** and **B.**, the triangle color indicates the value of its compactness score. The triangles outlined in red are triangles that would be filtered out using our sensitivity analysis algorithm. **C.** Histogram of compactness score for the two triangulations. The histogram outline in red is the result after filtering out the triangles shown in red in **A.** and **B.** respectively.

*6. It is also not clear to me how vertex pairing between configurations is ensured. Could you elaborate on this?*

**Response:** Thank you for addressing the potential confusion on this part. We have now added a demonstration of this vertex pairing in the new Fig S1C (see photo on the right also). In the experimental data, we manually match vertex (or marker point) pairs between the turgid and unturgid configuration by plotting them together in Matlab. Sometimes, when the two configurations are displaced in the experiment, the pairing is unclear. So, we add a global displacement of one set of marker points to aid the visual matching. For marker points that may have multiple potential matches, we further confirm their pairing by checking the raw data of the marker points. In ImageJ, we can visually confirm the location of the fluorescent bead before and after the deformation based on how the bead looks and its surrounding beads. If there is any uncertainty after visual confirmation in the raw data, we will not use that marker point position. In general, two fluorescent bead locations are an error’s length apart based on the software estimation, and also their known point spread functions. The vertex pairing explanation has been added to the Methods section and Section 1.2 in S1 Text.

*7. Currently, the method is tested only on synthetic data. A case study on real datasets would substantially strengthen the manuscript.*

**Response:** Thank you for this comment, we have now included our experimental data from *P. patens* cells. The results of this have been added to the last section in the Results (Line 440). The experimental results are also summarized in the new Figure 6. Additional clarification on the application of the method on the experimental data can be found in the Paragraph: “Marker point localization and automated triangulation” on Line 66. More details have also been added to Section 1.2 in S1 Text.

***Minor Comments***

-*Line 170: It would be instructive to evaluate the influence of different elastic potentials(besides Neo-Hookean) on bulk modulus estimation. Indeed, the Neo-Hookean model might not be the best-suited one to describe plant cell walls. Even if the ones considered here can be assumed isotropic, I wonder about their compressibility.* **Response:** Thank you for addressing this, we have also considered the idea of different material laws to describe plant cell walls. In the context of this paper and our lab’s previous works, our aim was to finish the story of the inference of tip-growing cell elasticity under the neo-Hookean compressible assumption. First, we want to explain the previous works and how we came to this assumption. Typically, models of plant growth (not just tip growth) use a hyperelastic model coming from the strain energy density function, or a fiber reinforced model which assumes anisotropy. It’s true that many plant models now use a fiber-reinforced anisotropic model [5, 6], but there has not been much published about fiber reinforcement in the field of tip growth specifically. Instead, other plant tissue and tip growth papers have used linear elasticity to describe the wall [7-10], for example using an extension of Hooke’s Law. Given the nonlinear stress-strain observed in plant cell walls, we chose to use the nonlinear neo-Hookean model instead [11]. The reason we don’t add a fiber reinforced assumption now, is because our focus is considering the wall area change, not thickness. We are aware that the cell wall is anisotropic along the normal direction, but for the 2D model we assume isotropy and compressibility along the 2-dimensions. This describes a transverse isotropic material, which has been seen in moss cell walls [12] (see photo to the right).

Roberts et al. 2012

- As a comparison, the constitutive law for a fiber-reinforced model with planar isotropic distribution would include the addition of the term: $\frac{1}{2}\eta\left( \frac{1}{2}\left( \lambda_{\theta}^{2}+\lambda_{s}^{2} \right)-1 \right)\lambda_{s}^{2}$ and $\frac{1}{2}\eta\left( \frac{1}{2}\left( \lambda_{\theta}^{2}+\lambda_{s}^{2} \right)-1 \right)\lambda_{\theta}^{2}$ to the Equations (4) and (5) in the main text, respectively [13]. In this case, $\eta$ is the material property describing the fiber-reinforcement, which affects compressibility. When strain is small, the equations at the leading order reduce to: $\sigma_{\theta}=\sigma_{s}=K_{h}\left( \epsilon_{s}+\epsilon_{\theta} \right)+\frac{1}{2}\eta\left( \epsilon_{s}+\epsilon_{\theta} \right)={(K}_{h}+\frac{1}{2}\eta)\left( \epsilon_{s}+\epsilon_{\theta} \right)$, where the $\epsilon$ terms are the elastic strains ($\epsilon=\lambda-1$). The linear hyperelastic model would be: $\sigma_{\theta}=\sigma_{s}=K_{h}(\epsilon_{s}+\epsilon_{\theta})$. Then we can observe that in the hyperelastic model, $\eta$ essentially gets absorbed into $K_{h}$. Therefore, the scale of our inference should not be drastically different given this additional assumption. If the cell wall is fully anisotropic, we agree that this will change our model. However, the inference of the elastic moduli should still be in the right range.
- *Line 182: Equations 9 and 10 may be replaced with those for bulk and shear moduli expressed as function of the geometrical descriptors and obtained by combining equations (Eqs. 5–8) .* **Response:** We have added this version of the equation as part of the original equations 9 and 10 (now equations 6 and 7 in the revised version).
- *Line 230: How is the tip position of the cell determined?* **Response:** The tip is determined by the maximum x-position of the wall outline. This has now been explained in the new Line 198 of the main text, and the new Fig S1 D. We have previously done testing on how the tip position’s affect by using the simulated cells. After perturbing the tip position slightly, the inferred results were qualitatively similar.
- *Fig. S4C: Indicate which curves correspond to circumferential and meridional stretches.* **Response:** Thank you, we have now indicated this on the curves (old Fig S4C, new Fig S6C).
- *Line 297: Null Poisson’s ratio hypothesis may be bold, given the material properties at stake.* **Response:** To clarify, we only used the assumption of null Poisson’s ratio in the test cases for the sphere-fitting method at the very beginning. This was to match what was used in Chevalier et al [14]. Before when we tested the sphere-fitting method and did not use Poisson’s ratio = 0, we obtained qualitatively similar results. Other viscous models have assumed Poisson’s ratio was 0 specifically at the tip [15], or have classified it as a free parameter in their model [9]. We have adopted a similar approach for the sphere-fitting, but we agree that this is a limitation of our test case. For all the simulation inferences using the main method, the limitation falls on the other material property µ. We discussed the difficulty in inferring µ in the Paragraph: “Instability of the bulk and shear modulus against small perturbations”, specifically on Line 183. Since we have fixed its value and gradient in relation to K, we setup µ so that it implies a constant Poisson’s ratio but not Poisson’s ratio = 0 (see the last paragraph of Section 1.6 in S1 Text). Once we have measured µ, the relation described on Line 162 comparing Poisson’s ratio, Youngs Modulus, K and µ, can be used to determine Poisson’s ratio. We have added more on the limitations of the inference of µ to the Discussion section in Line 559.
- *Lines 273–291 , Fig. 3: It would be valuable to test artificial surfaces lacking axial symmetry (e.g., by deforming the point cloud orthogonally to the main axis).* **Response:** We stated that the method is a way around the axisymmetric assumption, which we realize now may have been misleading. Our assumption is that the surface must be approximately axisymmetric so that the equations involving $\kappa_{s,\theta},\sigma_{s,\theta}$, and $\lambda_{s,\theta}$ are meaningful with the definition of the optimized circumferential and meridional directions. Therefore, we would not be able to test this on surfaces lacking axial symmetry. We have rewritten parts referencing any deviation from axial symmetry that may have been confusing.
- *Fig. 3D: The normalized displacement field noise appears high (∼ 10%). It would be informative to report noise level on the deformation gradient (F), in order to estimate the signal /noise ratio on the stretch estimation, central to the inference pipeline.* **Response:** Thank you for addressing this. From this, we were able to make additional connections between the noise level and F, numerically and analytically. Numerically, we looked at the error in the principal stretch ratios, $\lambda_{1}$ and $\lambda_{2}$, which are the eigenvalues of FF^T^. Analytically, we compared the theoretical upper limit of the error on F to the error on $\lambda_{1}\lambda_{2}$ : $\frac{\delta\left( \lambda_{1}\lambda_{2} \right)}{\lambda_{1}\lambda_{2}}=\frac{\delta\lambda_{1}}{\lambda_{1}}+\frac{\delta\lambda_{2}}{\lambda_{2}}\leq\frac{\delta\left\| F \right\|_{2}}{\left\| F \right\|_{2}}+\frac{\delta\left\| F \right\|_{2}}{\left\| F \right\|_{2}}=\frac{2\delta\left\| F \right\|_{2}}{\left\| F \right\|_{2}}$ (done similarly in Deng et al. 2022 [3]). In the “Triangle Error Analysis” section of S1 Text, we reported $\frac{\delta\left\| F \right\|_{2}}{\left\| F \right\|_{2}}$ in relation to a function we called $\Gamma|\delta|$. Therefore, our upper limit for each triangle is $2\Gamma|\delta|$, which we plotted in black in Figure 2 above. Numerically, we calculated the relative error of $\lambda_{1}\lambda_{2}$ using the value of $\lambda_{1}\lambda_{2}$ with and without noise (see Figure 2 above, red line). For all triangles and on average, the error is always below the theoretical upper limit (see Figure 2 above). We then estimated the upper limit of the displacement noise, $\delta$ and plotted it in blue. We saw that the signal to noise ratio results and theoretical upper bounds were consistent with findings that the large triangulations are less sensitive to noise. The large triangulation upper bound results are consistently lower than the other triangulations, especially at the tip. And they show the error mostly falling below the blue line in all noise cases (Figure 2A above), while the medium and small triangulation errors are equal or above. In summary, 10% noise is not as amplified in practice, while theory predicts larger magnification where errors interact in the worst way possible. We have added this result to Figure 3E, the new Fig S8, the Section “Origins of Noise” in the Methods on Line 305, and the Results Line 375.

**Figure 2: Area stretch ratio error is below theoretical limit and comparable to displacement noise.** For the three triangulation cases **A.** large, **B.** medium, and **C.** small, the relative error of $\lambda_{1}\lambda_{2}$ is shown in red. The theoretical upper limit of the error using the function $2\Gamma|\delta|$ is shown in black. For both values, the individual triangles and binned average are plotted (see legend). For each noise case, we estimated the upper limit of the displacement noise $|\delta|$ (blue line).

- *Lines 300–302: Origin of expressions for Y_side_ and Y_tip_ should be documented.* **Response:** Thank you, we have added this in Line 274.
- *Line 477: Maybe I missed the information, but how many samples did you take on the grid you mention on the (K_side_, K_tip_) space?* **Response:** We took the result from averaging 10 cells and then repeated this 50 times to reduce the randomness and obtain a smooth grid. This is explained in Section 1.7 of S1 Text.
- *Line 487: I find the term “false-positivity” ill-suited to the context, for there is no binary (true/false) results here. Wouldn’t the term “over-estimation” be more relevant?* **Response:** Thank you, we have removed the “false-positivity” term to avoid confusion. Instead, we have used the term “false gradient” to more clearly state the problem of inferring gradient results in cells with a ground truth constant elastic modulus. To avoid confusion on over/under-estimating the gradient itself versus the value of K_h_ at the side or tip, we did not use the term “over-estimation” to classify the phenomenon.
- *Fig. 7: Unusual color map orientation (lowest values at the top), this is a bit surprising.* **Response:** We have changed this.
- *Supp. Mat S1 : In Eq. 3, should the denominator be F^0^_2D_?* **Response:** Thank you, we have fixed this.
- *Section 1.4 refers to Fig. S2C but only two subpanels are in Fig. S2.* **Response:** Thank you, this was a typo as we meant to reference the old Fig S1 (now the new Fig S3).
- *Supp. Fig S7: Caption switches between tension and elastic stretch for panels B and C.* **Response:** Thank you, we have fixed this in the caption for the new Fig S9.
- *Sensitivity analysis is a notable strength; authors may elaborate further, e. g. , how triangle shape and size affect sensitivity. Key figures could be moved to the main text.* **Response:** Thank you, we have added more information on the triangle shape and sensitivity in the Methods section in Line 305 and in Figure 3E. As for the noise versus triangulation resolution, those results are summarized in Fig 7-8 of the Main Text.

***Personal option***

*The manuscript PCOMPBIOL-D-25-01862 proposed by Rholee Xu and coworkers presents a substantial work and I particularly enjoyed reading the developments of the sensitivity analysis proposed in Supp. Mat. S1 , as well as the noise versus resolution analysis. Nevertheless, in its present form, the manuscript does not — to my opinion —meet the publication standards of PLOS Computational Biology, primarily owing to three concerns: (i) The reliance on proprietary software and lack of open-source code. (ii) The absence of demonstration on experimental datasets. (iii) The reliability of the stereographic projection-based triangulation, which should be if not demonstrated at least discussed properly. Finally, a substantial re-writing effort should be consider to make the text more accessible to a wider audience. Addressing these points will greatly enhance the manuscript’s scientific value.*

**Response:** Thank you for your thorough comments and informed suggestions. This has helped us with the overall story of the manuscript, and the re-writing to make the text more accessible.

(i) As stated above in point (4), we have reworked the GitHub repository so that readers interested in our method have access to all the code used. This includes a demonstration on real experimental data, access to all the functions used to generate the results, and simulation data and code.
(ii) We have now added our experimental results. The demonstration of this is in the code repository and explained in parts in the Methods section and S1 Text. The focus of the paper is still on the method itself, but we have re-framed it to use the experimental data and results as motivation for the parameter sensitivity study.
(iii) As addressed in point (5) above, we demonstrated that the stereographic projection-based triangulation is more reliable than directly using the MATLAB function on the 3D marker points. We have seen its reliability to produce surface triangulations of different sizes, with generally compact triangles on many simulated cells. And for the experimental data, we only suggest the automation as a starting point for triangulation connectivity. The triangulation can be manually edited afterwards to ensure it matches the cell surface. To demonstrate this in the manuscript, we have included the Figure 1 shown here as the new Fig S2.

***Bibliography:***

- 1.	Abenza, J.F., et al., *Wall mechanics and exocytosis define the shape of growth domains in fission yeast.* Nat Commun, 2015. **6**: p. 8400.
- 2.	Davi, V., et al., *Systematic mapping of cell wall mechanics in the regulation of cell morphogenesis.* Proc Natl Acad Sci U S A, 2019. **116**(28): p. 13833-13838.
- 3.	Deng, Y., et al., *Inferring relative surface elastic moduli in thin-wall models of single cells.* The European Physical Journal Plus, 2022. **137**(8).
- 4.	Ohairwe, M.E., B.D. Zivanovic, and E.R. Rojas, *A fitness landscape instability governs the morphological diversity of tip-growing cells.* Cell Rep, 2024. **43**(4): p. 113961.
- 5.	Huang, R., A.A. Becker, and I.A. Jones, *A finite strain fibre-reinforced viscoelasto-viscoplastic model of plant cell wall growth.* Journal of Engineering Mathematics, 2015. **95**(1): p. 121-154.
- 6.	Chakraborty, J., J. Luo, and R.J. Dyson, *Lockhart with a twist: modelling cellulose microfibril deposition and reorientation reveals twisting plant cell growth mechanisms.* Journal of Theoretical Biology, 2021. **525**: p. 110736.
- 7.	Hervieux, N., et al., *A mechanical feedback restricts sepal growth and shape in Arabidopsis.* Current biology, 2016. **26**(8): p. 1019-1028.
- 8.	Armezzani, A., et al., *Transcriptional induction of cell wall remodelling genes is coupled to microtubule-driven growth isotropy at the shoot apex in Arabidopsis.* Development, 2018. **145**(11): p. dev162255.
- 9.	Rojas, E.R., S. Hotton, and J. Dumais, *Chemically mediated mechanical expansion of the pollen tube cell wall.* Biophys J, 2011. **101**(8): p. 1844-53.
- 10.	Fayant, P., et al., *Finite element model of polar growth in pollen tubes.* Plant Cell, 2010. **22**(8): p. 2579-93.
- 11.	Zhang, Y., et al., *Molecular insights into the complex mechanics of plant epidermal cell walls.* Science, 2021. **372**(6543): p. 706-711.
- 12.	Roberts, A.W., E.M. Roberts, and C.H. Haigler, *Moss cell walls: structure and biosynthesis.* Front Plant Sci, 2012. **3**: p. 166.
- 13.	Holzapfel, G.A. and R.W. Ogden, *Biomechanical modelling at the molecular, cellular and tissue levels*. Vol. 508. 2009: Springer Science & Business Media.
- 14.	Chevalier, L., et al., *Cell wall dynamics stabilize tip growth in a filamentous fungus.* PLoS Biology, 2023. **21**(1): p. e3001981.
- 15.	Rabille, H., et al., *The brown algal mode of tip growth: Keeping stress under control.* PLoS Biol, 2019. **17**(1): p. e2005258.
