# Supplementary material for: A surface morphology-based inference method for the cell wall elasticity profile in tip-growing cells
Source: PLoS Comput Biol. 2026 Apr 2;22(4):e1013532. doi: 10.1371/journal.pcbi.1013532 (PMC13082730; doi:10.1371/journal.pcbi.1013532)
Supplement: Attachment — Submitted filename: Rebuttal Letter_2nd round.docx [file pcbi.1013532.s023.docx]

We would like to thank Reviewer 1 and Reviewer 2 for looking over and approving our major revisions. We have responded to both reviewers’ most recent comments below. Changes in the main text have been made in red.

***Reviewer #1****: I thank the authors for taking the time to answer my comments. I am satisfied with their answers. I have a final comment regarding the new Fig. 6. The stresses (Fig. 6D, second row) should be in the ratio 2:1 (circumferential: meridional) at the base of the tip (local angle = 90 deg.). For both cell types (especially the chloronema), the experimental data deviate from this ratio. Could the authors explain why? I could not found an explanation in the main text.*

**Response:** Thank you for this comment as we did not address this in the main text. This is because the meridional curvature measured at the side of the cell does not reach 0 strictly. Our current $\kappa_{s}$ not reaching 0 at the side region is due to the fact that the current data coverage in the last bin covers regions with local normals between 80-90 degrees away from the long-axis direction. In addition, the current data set did not cover a larger portion of the flat region of the cell. When there is large-enough flat-region data, the mean of $\kappa_{s}$ might reach zero, which could then recover the 2:1 ratio. We have added this explanation to the main text in the new Line 450.

***Reviewer #2:*** *Dear Authors,*

*Thank you for this revisited version of your manuscript and the significative amount of work and effort you put into it. It seems that most of my main initial concerns have been addressed and I am please to support the acceptance of your manuscript.*

*I however suggest two (very) minors (and optional) upgrades :*

*1. Maybe I missed it, but I think the github repository is not explicitly mentioned in the main text. It would be worth having a "Code availability" subsection, at the end of the "Methods" section, pointing directly to this repository. Or maybe this is something handled by the editorial team directly ?*

**Response:** Thank you for this suggestion as we did not explicitly mention the GitHub link in the main text. In the original portal submission, we were prompted for a “Code and Data Availability Statement” which we included. We think it would be best if the link was mentioned in this section, which we will make sure is included in the final document.

*2. Concerning the code repository, it seems to serve its purpose and the detailed README file is appreciated. However, a more throughout description of the installation procedure mentioning the required softwares and where to get them (inserted at the beginning of this README) would certainly help the interested readers to use the proposed code.*

**Response:** Thank you for suggesting this. We have added an additional section called “Software installation” at the start of the README file in GitHub to describe the installation of the required software.
